# A Population Approach to Transportation Planning: Reducing Exposure to Motor-Vehicles

**DOI:** 10.1155/2013/916460

**Published:** 2013-06-13

**Authors:** Daniel Fuller, Patrick Morency

**Affiliations:** ^1^Department of Community Health and Epidemiology, University of Saskatchewan, Health Sciences Building, 107 Wiggins Road, Saskatoon, SK, Canada S7N 5E5; ^2^Direction de Santé Publique de Montréal, Université de Montréal, 1301, rue Sherbrooke Est, Montréal, QUE, Canada H2L 1M3; ^3^Département de Médecine Sociale et Préventive, Université de Montréal, 1301, rue Sherbrooke Est, Montréal, QUE, Canada H2L 1M3

## Abstract

Transportation planning and public health have important historical roots. To address common challenges, including road traffic fatalities, integration of theories and methods from both disciplines is required. This paper presents an overview of Geoffrey Rose's strategy of preventive medicine applied to road traffic fatalities. One of the basic principles of Rose's strategy is that a large number of people exposed to a small risk can generate more cases than a small number exposed to a high risk. Thus, interventions should address the large number of people exposed to the fundamental causes of diseases. Exposure to moving vehicles could be considered a fundamental cause of road traffic deaths and injuries. A global reduction in the amount of kilometers driven would result in a reduction of the likelihood of collisions for all road users. Public health and transportation research must critically appraise their practice and engage in informed dialogue with the objective of improving mobility and productivity while simultaneously reducing the public health burden of road deaths and injuries.

## 1. Introduction

Transportation planning and public health have important historical connections [1]. In the early 20th century transportation planning and public health held similar objectives. In 1909 Marsh wrote, “city planning is the adaptation of a city to its proper function. This conception can be indefinitely expanded but its significance will be appreciated if we admit that no city is more healthy than the highest death rate in any ward or block and that no city is more beautiful than its most unsightly tenement (p.27)” [2]. Since the early 20th century, the relationship between transportation planning and public health has waxed and waned [1, 3]. There is growing recognition that integration of theories and methods from each discipline is beneficial for advancing research and practice [4]. 

Of particular relevance to both transportation planning and public health are injuries due to traffic collisions. Worldwide, traffic collisions are one of the leading causes of death among youth and young adults [5]. A number of intervention strategies including black spot analyses are used in transportation planning to reduce road fatalities. There is ongoing debate in the transportation planning literature that this type of intervention may have limited effectiveness in reducing road fatalities [6–8]. New approaches are needed to reduce the burden of road traffic fatalities. 

Given the need to reconnect transportation planning and public health, this paper will present Rose's strategy of preventive medicine [9, 10], a promising intervention approach from public health. The approach is applied to the analysis and prevention of road traffic fatalities. 

## 2. The Prevention Paradox

The prevention paradox states that “a preventive measure which brings much benefit to the population offers little to each participating individual (p.38)” [10]. The prevention paradox is the basis for Rose's strategy of preventive medicine. The prevention paradox is based on an important distinction between an individual case and the incidence of cases in a population. A case is defined as an episode of disorder, illness, or injury affecting an individual, while incidence is defined as “the number of cases of a disease that come into being during a specific time period (p.44)” [11]. The basic principle of Geoffrey Rose's strategy of preventive medicine is that the causes of cases (i.e., why a specific individual is involved in a collision) are different from the causes of population incidence (i.e., why there are over 3 million collisions per year in the United States) [9]. 

## 3. The Majority of Cases Occur in Individuals at Average Risk

Building from the prevention paradox, the primary concept of Rose's strategy is that the majority of cases do not occur in individuals at high risk. Thus, “a large number of people exposed to a small risk may generate many more cases than a small number exposed to a high risk (p.59)” [9]. The recent study by Beck [12] provides an example of this principle related to road traffic fatalities. 

**Figure 1:**
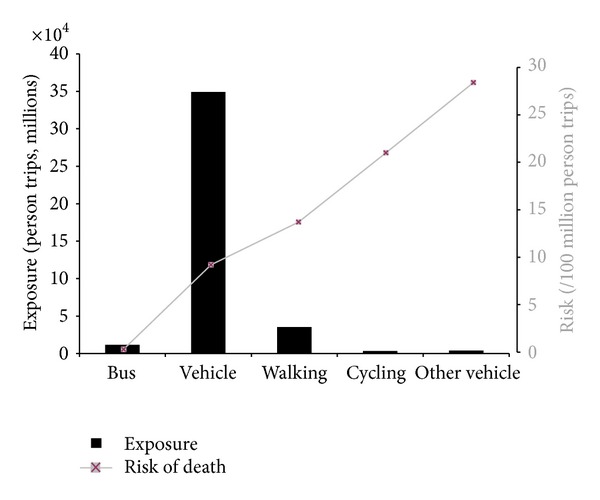
Theoretical distribution of the continuum of risk for all road traffic users. Note: figure adapted from Beck et al. [12].

Beck et al. show that pedestrians (13.7 deaths per 100,000 person trips), cyclists (21.0 deaths per 100,000 person trips), and motorcyclists (536.6 deaths per 100,000 person trips) are at higher risk of a fatal collision than motor vehicle users. Despite their higher risk, pedestrians, cyclists, and motorcyclists represent only 20.5% of those killed in traffic collisions.  Figure 1 shows that in the USA the majority of trips (86%) and therefore exposure to causes of collisions are greater for motor vehicle users. Thus, the majority of those killed (76.6%) are motor vehicle users who are, on average, at a lower risk (9.2 deaths per 100,000 person trips) than other road users except public transit users. 

## 4. Acting on Causes of Incidence

For interventions to be effective in reducing the total number of road deaths and injury, they must target fundamental causes [13] (i.e., causes of incidence), which would result in a global reduction in exposure to a fundamental cause for the entire population. According to the strategy of preventive medicine road traffic fatalities can be neither understood nor properly controlled if high-risk road users are thought to constitute the entire problem. Policies and practices that attempt to prevent traffic fatalities must consider all modes of transportation because they all contribute to the total number of deaths (Figure 1) [14, 15].

**Figure 2:**
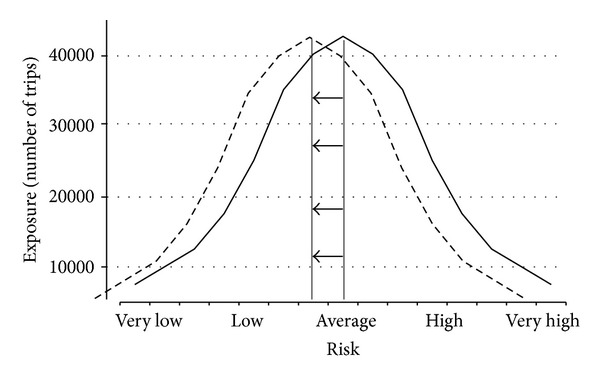
Theoretical distribution of the exposure to risk of road death and injury, showing a reduction in the average exposure for the entire continuum of risk (dotted line). Note: although this figure is theoretical, recent researches estimated the potential reduction in injury risk at intersections following reduction in traffic volume [17, 38] or a modal shift toward walking and cycling [18].

In transportation research, the volume and speed of motor vehicle traffic could be considered two of the fundamental causes of collisions, for all road users [16, 17]. Throughout the paper, we consider exposure to moving vehicles as a fundamental cause of road traffic deaths and injuries. In this example, a global reduction in the amount of kilometers driven would result in a reduction of the likelihood of collisions for all road users, a left shift in the risk of road death and injury (Figure 2) [18, 19]. For all road users, including pedestrians and cyclists, a reduction in traffic volume contributes to a lower risk of injury and death. Significant public health benefits could be achieved through macrolevel interventions that influence the level of exposure to traffic volumes for all road users, for example, land use and transportation policies that encourage using safer transportation modes (e.g., public transit) and area-wide traffic calming schemes covering whole metropolitan areas, among others. In the USA, recent reductions of traffic volume were associated with a reduction in the total number of road fatalities [20]. Potential mechanisms for this reduction include an economic recession and higher gas prices. 

## 5. Acceptability of Change

Rose's perspective is one way to critically appraise the challenge of reducing the overall burden of collisions while maintaining the benefits of effective mobility. However, it remains theoretical. The implementation of interventions supported by Rose's strategy requires a dialogue between researchers, planners, policy makers, and the public. Throughout this discussion we must recall that the primary function of roadways is not to cause collisions but rather the mobility and productivity of the population [21]. Because roadways have multiple functions, policy makers, planners, health officials, and the public may disagree on how to balance road safety with other competing functions. As Rose states, “if a problem is common and has been around for a long time, then people come to accept it even if it is large: it is the exceptional or new which causes alarm. The toll of deaths from road traffic accidents vastly exceeds that from crashing aero planes, but only the latter lead to public health inquiries (p.57)” [9]. As the previous quote suggests, researchers must critically appraise their work and dialogue with policy makers and the public about complex transportation challenges in order to achieve an acceptable balance between the need for mobility and its consequences [22–24]. Ideally, interventions will both improve mobility and reduce the public health burden of road traffic injuries. In particular researchers must be conscious of the potential influence of structural global economic forces lead by major car and oil companies in influencing road collisions research and discourse [21, 25–27]. 

## 6. An Example: Road Traffic Collisions, Injuries, and Deaths

Beck et al. state, “our findings suggest that a shift from passenger vehicle travel (lower risk) to nonmotorized travel (higher risk) could result in an overall increase in the numbers of people killed in traffic… measures that prevent crashes and injuries for pedestrians and bicyclists are needed, especially given the recent focus on increasing physical activity through active travel (p.216)” [12]. The discussion by Beck et al. suggests that the high-risk approach is a more acceptable intervention than a population approach, which would reduce exposure to fundamental causes, volume of motor vehicles in our example, for all road users. However, in general, interventions targeting high-risk groups (e.g., walkers and cyclists) have limited population wide benefit for injury prevention. Targeting the causes of incidence to reduce exposure to motor vehicle volume for all road users, the population approach, has the potential for a greater reduction in the total number of transportation fatalities in a population than the high-risk approach. 

Reanalyzing the data from Beck et al. compares a population approach and a high-risk approach targeting pedestrians and cyclists. In the USA, between 1999 and 2003, a 25% reduction in the number of pedestrian and cyclist fatalities would have resulted in 1386 fewer deaths (1212 for pedestrians, 174 for cyclists). A greater number of lives would have been saved by a 4% reduction in the fatal risk for all road users (Table 1). Table 1 estimates the hypothesized effect of decreasing fatal risk over the entire population of road users. It shows that a 10% and 15% reduction in the fatality risk for all road users would result in a reduction of 4213 and 6320 cases, respectively. These scenarios would benefit motor vehicle occupants but also pedestrians and cyclists. In urban settings, a risk reduction of 15% could be achieved through area-wide traffic calming interventions [28]. 

Reducing the risk of road death and injury by intervening to reduce the level of exposure to traffic volumes, or other fundamental causes, has many consequences. First, the potential reduction in road injuries and deaths is much larger than with high-risk approaches. Second, a population-wide prevention strategy is of benefit to high-risk road users, whatever the criteria used to define “high risk” (e.g., transportation mode, alcohol consumption, age group). Third, it may also reduce other externalities including pollution, noise, physical inactivity of fundamental causes such as traffic volume [21, 29]. 

**Figure 3:**
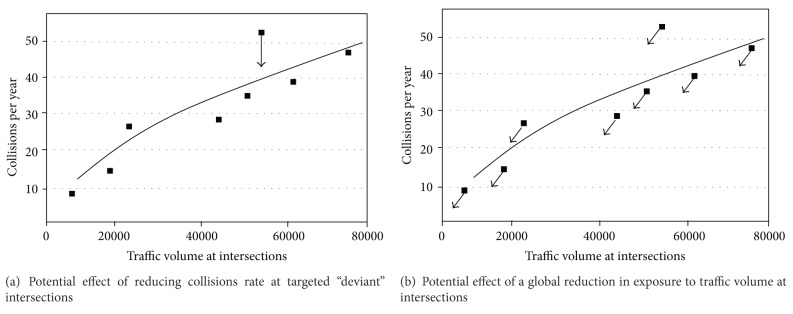
Approaches to address safety at intersections using the traditional high-risk approach (a) and the population approach (b). Note: the figure is adapted from: US Department of transportation, Federal Highway Administration [32]. Signalized intersection: Informational guide. Publication no. FHWA-HRT-04-091. Arrows represent the hypothetical change in the number of collisions per year associated with high-risk (a) and population (b) approaches.

For decades, the strong relationship between traffic volume and road traffic injuries or death has been reported, at the street, city, region, or country levels [30, 31]. For all intersections from a road network, Safety Performance Functions show that the expected number of collisions at intersections increases as traffic volume increases, though this relationship is not linear (Figure 3). The traditional intervention strategy, recommended by the US Department of Transportation [32] is to return deviant intersections to within an acceptable range of the average collisions rate at comparable intersections. As seen in Figure 3(a), this targeted intervention approach aims to move the deviant intersections closer to the best fit regression line. This is an example of a high-risk approach that does not address the entire population of intersections or injured individuals. In Montreal, for example, only 4% of pedestrian injuries occurred at 22 identified black spot intersections, whereas the remaining 96% of pedestrians were injured at more than 3500 different crash sites, none of which were “black spots” [8].

The population strategy acknowledges that interventions should address the fundamental causes of road deaths and injuries, in our example, the level of exposure to moving vehicles over the entire population, and for all intersections. The population approach would ask whether it was possible to globally reduce the exposure to traffic volume (Figure 3(b)) and subsequently reduce the risk of death and injury at any given intersection for all road users. As the arrows show in Figure 3(b), the traffic volume and subsequent risk are reduced at every intersection. In theory, it would result in a greater reduction of the number of injured road users than targeting deviant streets and intersections. 

## 7. A Caveat

This paper presents a simplified version of Rose's strategy applied to road collisions. The intent was not to present the theory in its entirety. Rather we present a promising approach to think about road traffic fatalities. A detailed reading of Rose's work [9, 10] will provide more precision and help clarify interested readers understanding. It should be noted that Rose's ideas are not without considerable debate in public health [33–35]. For example, modeling studies suggest that Rose's approach is generally more cost effective; however, in certain cases a high risk approach can have better cost/benefit ratios [33]. For example, recent studies suggest that when compared with other roads, streets with cycle tracks have a lower risk of injury for cyclists [36, 37]. As well, the potential of Rose's strategy for reducing social inequities in health in general [34, 35] and in particular road fatalities for those residing in low income areas is still debated. A vulnerable population approach may be the most acceptable when attempting to act on social inequities; however, a recent study has shown that reducing traffic volume at all intersections of a Canadian city would be most beneficial in poorer neighborhoods [38].

## 8. Conclusion

Transportation planning and public health have important historical roots. To address common challenges, including road traffic fatalities, integration of theories and methods from both disciplines is required. Geoffrey Rose's strategy of preventive medicine made an important theoretical contribution to public health and has applications to transportation. The present paper demonstrated Rose's theory and provided examples drawn from transportation research. This paper allowed for both public health and transportation research to critically appraise their practice and engage in informed dialogue with the objective of improving mobility and productivity while simultaneously reducing the public health burden of road deaths and injuries.

## Figures and Tables

**Table 1:** Estimated reduction in risk resulting from a 15%, 10% and 4% shift in the risk distribution for motor vehicle crash injury rates for all modes in the United States*.

	Person Trips (million)	Fatal risk per 100 million person trips	Number of fatal injuries			
			Status quo	Scenario of risk reduction		
				−15%	−10%	−4%
Vehicle	349125	9.25	32283	−4842	−3228	−1291
Walking	35366	13.7	4846	−727	−485	−194
Bus	11458	0.35	40	−6	−4	−2
Other vehicle	4068	28.42	1156	−173	−116	−46
Cycling	3314	20.97	695	−104	−70	−28
Motorcycle	580	536.55	3112	−467	−311	−124

Total	403,911	10.43	42132	−6320	−4213	−1685

*Note. Table adapted from [12].
